
JOURNAL INFORMATION
==============================
NLM Title Abbreviation: Hemasphere
Journal ID: Hemasphere
NLM Journal ID: 101740619
Journal ID: HEM3

HemaSphere

EISSN: 2572-9241
Publisher: Wiley

ARTICLE INFORMATION
==============================
PMCID: PMC12331864
DOI: 10.1002/hem3.70203
Article ID: HEM370203
Article version: 1
Subjects: Issue Information, Issue Information

Issue Information

Electronic publication date: 2025 Aug 7
Publication date: 2025 Aug
Volume: 9
Issue: 8
Electronic Location ID: e70203
Copyright: © 2025 The Authors. HemaSphere published by John Wiley & Sons Ltd. on behalf of European Hematology Association.
License: This is an open access article under the terms of the http://creativecommons.org/licenses/by/4.0/ License, which permits use, distribution and reproduction in any medium, provided the original work is properly cited.
License URL: https://creativecommons.org/licenses/by/4.0/

Figure count: 0
Table count: 0
Page count: 4

source-schema-version-number: 2.0
cover-date: August 2025
details-of-publishers-convertor: Converter:WILEY_ML3GV2_TO_JATSPMC version:6.5.9 mode:remove_FC converted:08.08.2025

==============================

### HemaSphere

Vol. 9 - Issue 8

August 2025

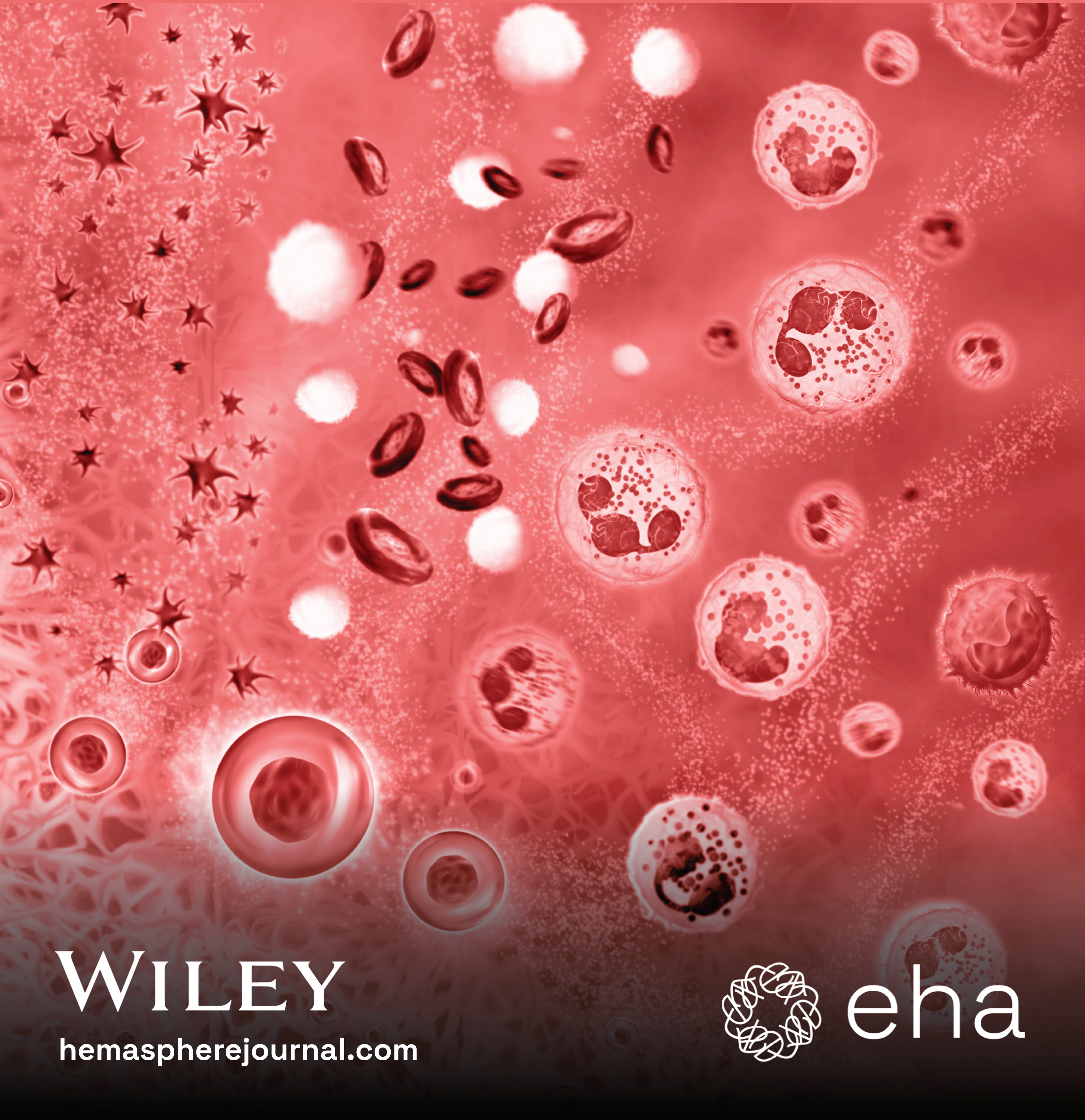

WILEY

hemaspherejournal.com eha

HemaSphere

### Aims and Scope

*HemaSphere* publishes results of highly relevant basic, translational, and clinical research in hematology. We are especially looking for strong studies reporting novel findings that are of high impact to the field of hematology.

Review articles and guideline articles provide clear overviews and discussions of new developments as well as recommendations for patient care.

*HemaSphere* is the premier hematology information resource, which is enhanced by the HemaTopics and HemaPolicy sections of the journal. These sections provide insightful discussions on all aspects related to hematology, including summaries of important articles, reports of new therapies, discussions on European policy, and other hematology news items. *HemaSphere* is guided by Editor in Chief Jan Cools and Deputy Editor in Chief Claire Harrison. The Editors and Editorial Board of *HemaSphere* are international research and clinical leaders, representing a diverse range of areas of hematologic expertise.

### Open Access Licensing and Copyright

*HemaSphere* is an Open Access journal: authors of accepted articles pay an article publication charge, and their articles are published under a Creative Commons license.

Certain funders mandate a particular type of CC license is used. All other authors can choose between the CC BY-NC and CC BY-NC-ND Creative Commons Licenses.

For more information about the licenses offered by this journal, please visit Wiley Author Services.

### Disclaimer

The Publisher, European Hematology Association, and Editors cannot be held responsible for any errors in or any consequences arising from the use of information contained in this journal. The views and opinions expressed do not necessarily reflect those of the Publisher, European Hematology Association, or Editors, neither does the publication of advertisements constitute any endorsement by the Publisher, European Hematology Association, Editors, or Authors of the products advertised. Wiley Open Access articles posted to repositories or websites are without warranty from Wiley of any kind, either express or implied, including, but not limited to, warranties of merchantability, fitness for a particular purpose, or non-infringement. To the fullest extent permitted by law Wiley disclaims all liability for any loss or damage arising out of, or in connection with, the use of or inability to use the content.

HemaSphere eha

**Editorial Board**

**Editor in Chief**
**Professor Jan Cools**
*VIB-KU Leuven Center for Cancer Biology, Leuven, Belgium*

**Deputy Editor in Chief**
**Professor Claire Harrison**
*Guy's and St Thomas' NHS Foundation Trust, London, UK*

**Associate Editors**
Jeroen Eikenboom, *Leiden University Medical Centre, Leiden, The Netherlands*
Simone Ferrero, *University of Torino, Italy*
Adele Fielding, *University of York, Hull York Medical School, York, UK*
Simon Hallam, *St. Bartholomew's Hospital, Barts Health NHS Trust, London, UK*
Steffen Koschmieder, *University Medical Center RWTH, Aachen, Germany*
Marek Mraz, *CEITEC Masaryk University and University Hospital Brno, Czech Republic*
Paula Rodríguez Otero, *Clínica Universidad de Navarra, Spain*
Jürg Schwaller, *University Hospital Basel, Switzerland*
Francesca Vinchi, *Iron Research Laboratory - New York Blood Center, & Weill Cornell Medicine - Pathology and Laboratory Medicine, New York, USA*

**Scientific Editors**
Charles de Bock, *Children's Cancer Institute, Sydney, Australia*
Stephen Hibbs, *Queen Mary University of London, UK*
Mandy Lauw, *Erasmus University MC, Rotterdam, The Netherlands*
Lauren Merz, *University of Michigan, Ann Arbor, Michigan, United States*
Edeghonghon Olayemi, *University of Ghana Medical School, Accra, Ghana*

**Statistics Editor**
Robert Hills, *University of Oxford, UK*

**Editorial Manager, European Hematology Association**
Jessica Mastrodomenico, *EHA/HemaSphere, The Hague, The Netherlands*

**Editorial Board**
Frederic Baron, *University of Liège, GIGA Institute, Liège, Belgium*
Grzegorz Basak, *Medical University of Warsaw, Poland*
Andrea Biondi, *University of Milano-Bicocca, Italy*
Jonathan Bond, *University College Dublin, Ireland*
Dominique Bonnet, *The Francis Crick Institute, London, UK*
Arndt Borkhardt, *University of Düsseldorf, Germany*
Monika Brüggemann, *University Hospital Schleswig-Holstein, Kiel, Germany*
David Bryder, *Lund University, Sweden*
Ryan Cassaday, *Fred Hutchinson Cancer Center, Seattle, Washington, USA*
Giancarlo Castaman, *Azienda Ospedaliera Universitaria Careggi, Florence, Italy*
Anna Castleton, *The Christie Hospital NHS Foundation Trust, Manchester, UK*
Nicholas Chiorazzi, *Northwell Health, Manhasset, New York, USA*
Emmanuelle Clappier, *Hôpital Saint-Louis, Paris, France*
Paolo Corradini, *Università degli Studi di Milano, Italy*
Lucia De Franceschi, *University of Verona, Italy*
Kim De Keersmaecker, *University of Leuven, Belgium*
Marianne De Montalembert, *Hôpital Universitaire Necker-Enfants Malades, Paris, France*
Michel Delforge, *Leuven Cancer Institute, Leuven, Belgium*

Catherine Diefenbach, *Perlmutter Cancer Center, New York, USA*
Carlos Fernández De Larrea, *Hospital Clínic Barcelona, Spain*
Francesca Gay, *University of Turin, Italy*
Paolo Ghia, *Università Vita Salute San Raffaele, Milan, Italy*
Domenico Girelli, *Universita degli Studi di Verona, Italy*
Florian Grebien, *University of Veterinary Medicine Vienna, Austria*
Hildegard Greinix, *Medical University of Graz, Austria*
Kirsten Grønbæk, *Rigshospitalet, Copenhagen, Denmark*
Iqbal Hamza, *University of Maryland School of Medicine, Baltimore, Maryland, USA*
Florian Heidel, *University Medical Center Greifswald, Germany*
Olaf Heidenreich, *Princess Máxima Center, Utrecht, The Netherlands*
Brian Huntly, *University of Cambridge, UK*
Elaine Jaffe, *National Cancer Institute, Bethesda, Maryland, USA*
Mats Jerkeman, *Lund University, Sweden*
Efstathios Kastritis, *National and Kapodistrian University of Athens, Greece*
Léon Kautz, *Inserm, Toulouse, France*
David Kent, *University of York, UK*
Ulf Klein, *University of Leeds, UK*
Joost Kluiver, *Universitair Medisch Centrum Groningen, The Netherlands*
Daniela Krause, *University of Frankfurt, Germany*
Nicolaus Kröger, *University Hospital Hamburg-Eppendorf, Germany*
Steven Lane, *QIMR Berghofer Medical Research Institute, Australia*
Frank Leebeek, *Erasmus MC Cancer Institute, Rotterdam, The Netherlands*
Florent Malard, *INSERM, Saint-Antoine Hospital, Paris, France*
Maria-Victoria Mateos, *University of Salamanca, Spain*
Francesco Maura, *Memorial Sloan Kettering Cancer Center, New York, USA*
Simon Mendez-Ferrer, *University of Cambridge, UK*
Thomas Mercher, *Gustave Roussy Institute, Villejuif, France*
Martina Muckenthaler, *Heidelberg University Hospital, Heidelberg, Germany*
Cesar Nombela Arrieta, *University Hospital Zurich, Switzerland*
James O'Donnell, *Royal College of Surgeons in Ireland, Dublin, Ireland*
Kostas Pantopoulos, *McGill University, Montreal, Quebec, Canada*
Jakob Passweg, *University of Basel, Switzerland*
Heike Pfeifer, *University Hospital Frankfurt, Germany*
Marc Raab, *Heidelberg University Hospital, Heidelberg, Germany*
Marc Raaijmakers, *Erasmus Medical Center Cancer Institute, Rotterdam, The Netherlands*
Alejo Rodriguez-Fratelli, *Institute for Research in Biomedicine, Barcelona, Spain*
Richard Rosenquist, *Karolinska Institutet, Stockholm, Sweden*
Deepa Sampath, *MD Anderson Cancer Center, Houston, Texas, USA*
Rebekka Schneider, *Erasmus MC, Rotterdam, The Netherlands*
Marie Scully, *University College London Hospitals, NHS Foundation Trust, London, UK*
Kostas Stamatopoulos, *CERTH, Centre for Research and Technology Hellas, Thessaloniki, Greece*
Anna Maria Sureda Balari, *Institut Català d'Oncologia, Barcelona, Spain*
Evangelos Terpos, *National and Kapodistrian University of Athens, Greece*
Alexandre Theocharides, *University Hospital Zürich, Switzerland*
Jan Trka, *University of Prague, Czech Republic*
Karen Vanhoorelbeke, *KU Leuven, Laboratory for Thrombosis Research, Kortrijk, Belgium*
Carlo Visco, *University of Verona, Italy*
Clive Zent, *University of Rochester Medical Center, Rochester, New York, USA*
